# 

**Published:** 1899-08-26

**Authors:** 


### NOTICE TO CORRESPONDENTS.

**All MS., Letters, Books for Review, and other matters intended for the Editor should be addressed The Editor, "The Hospital," The Hospital Building, 28 & 29, Southampton Street, Strand, W.C.**
**The Editor cannot undertake to return rejected MS., even when accompanied by stamped directed envelope.**

**Notice to Advertisers and Subscribers.**
All Advertisements, Orders for Copies of The Hospital, and Business Communications should be addressed to **THE MANAGER (Not to the Editor)**, The Hospital, The Hospital Building, 28 & 29, Southampton Street, Strand, London, W.C.
The Cost of Subscription, post free, is as follows:—Per week, 2½d.; per quarter, 2s. 8d.; per half-year, 5s. 3d.; per annum, 10s. 6d. Foreign:—Per annum, 15s. 2d., payable, in all cases, in advance.

**NOTICE.—Vol. XXV. of "The Hospital" (October, 1898, to March, 1899), in handsome cloth binding, is now on sale at the Office of this Journal, price 7s. 6d. Cases for binding the Numbers contained in this Volume 1s. 6d. Index to Vol. XXV., 2½d., post free.**
**The Monthly Part for September will be ready on September 1. Price 6d., by post 8d.**

### Scraps and Gleanings.

The new memorial ward for children at the Clayton Hospital, which is to contain 24 to 28 cots, is now in progress of erection.

Up to the present date the total expenditure on the alterations at the Devon and Exeter Hospital has been upwards of £30,000.

At Armstrong's great shipbuilding works a sum of £2,000 is yearly collected on behalf of the Newcastle Hospital Sunday Fund.

The amount of workpeoples' contributions for this year to the Huddersfield Infirmary has reached the satisfactory total of £1,124 odd, being an increase over last year's contributions from the same source of upwards of £70.

At the meeting of the Mirfield and Liversedge Joint Hospital Board a resolution was passed that the Board take the necessary steps for the provision of hospital accommodation for the treatment of cases of typhoid fever in addition to scarlet fever.

At the quarterly court of the Devon and Exeter Hospital, the following resolution was carried: "That no patient or his friends shall pay whole or any part of a recommendation for admittance to the hospital, or such recommendation shall be forfeited."

### The Student of Medicine.

### APPOINTMENTS.

I. Aird, M.B., Ch.B.Edin., appointed Assistant Surgeon to the Liverpool Dispensaries. W. A. Clayton, L.R.C.P., L.R.C.S.Edin., L F.P.S.Glasg., appointed Medical Officer for the Sharlston and Crofton Districts of the Wakefield Union. C. J. A. Coates, L.R.C.P., L.R.C.S.Edin., appointed Medical Officer for the Tenth District of the Wycombe Union. J. Dalebrook, M.A.Oxon., M.R.C.S.Eng., L.R.C.P.Lond., appointed Second Assistant Medical Officer at the St. John's Road Workhouse and Infirmary of the Parish of St. Mary, Islington. A. M. Dodd, M.R.C.S.Eng., L.R.C.P.Lond., appointed Assistant Surgeon to the Liverpool Dispensaries R. B. Gass, M.B., C.M.Edin., appointed Medical Officer of Health for the Heysham Urban District. E. S. Gorman, M.B., B.Ch., B.A.O.R.U.I., appointed Assistant Resident Medical Officer to the Birmingham Workhouse Infirmary. T. G. Hodson, M.D., appointed Medical Officer for the Fourth District of the South Stoneham Union. W. P. Meldrum, M.B.. Ch.B. B.Sc.Edin., appointed Assistant Surgeon to the Liverpool Dispensaries. C. K. Millard, M.D., D.Sc. (Public Health) appointed Medical Officer of Health for the Borough of Burton-on-Trent. R. Moreton, M.R.C.S., L.R.C.P., appointed Medical Officer for the Wymondham District of the Melton Mowbray Union. F. G. Proudfoot, M.A.St.And., M.B., C.M.Edin., appointed District Medical Officer of the Oxford Incorporation. R. Storr, L.R.C.P., L.R.C S.Edin., L.F.P.S.Glasg., appointed Medical Officer for the Thorncombe District of the Beaminster Union. H. M. B. Stratford, M.R.C.S.Eng., L.R.C.P.Lond., appointed Assistant House Surgeon to the Hereford General Dispensary.

